# Supplementary material for: Fluid and solute transport by cells and a model of systemic circulation
Source: PLoS Comput Biol. 2025 Apr 21;21(4):e1012935. doi: 10.1371/journal.pcbi.1012935 (PMC12040233; doi:10.1371/journal.pcbi.1012935)
Supplement: S1 Table — Parameter values and sources for tissue-level and systems-level fluid transport models. (PDF) [file pcbi.1012935.s002.pdf]

Supplementary Table

Yufei Wu [1,2], Morgan A. Benson [1,2], Sean X. Sun [1,2,3,*]

[1]Department of Mechanical Engineering, Johns Hopkins University, Baltimore, Maryland, USA
[2]Institute for NanoBioTechnology, Johns Hopkins University, Baltimore, Maryland, USA
[3]Center for Cell Dynamics, Johns Hopkins School of Medicine, Baltimore, Maryland, USA

[*]To whom correspondence should be addressed; E-mail: ssun@jhu.edu.

Supplementary Tables

Table 1: Glossary of model variables and their numerical estimates for the pumping element. See text for references.

Symbol	Description	Values	Source
D	Diffusion coefficient of ions, e.g. K^+ (m^2/s)	2×10^{-9}	[3, 4]
γ	Active ion transport coefficient (m/s)	2.8×10^{-9}	[1, 6, 7]
β	Passive ion transport coefficient (m/s)	2.6×10^{-4}	[1, 6, 7]
α	rate constant of water transport ($m \cdot s^{-1} \cdot Pa^{-1}$)	10^{-9}	[1]
G	Energy input of active ion pumping (kJ/mol)	30	[1]
L	Thickness of renal epithelial (endothelial) tissue (m)	10^{-5}	-
m	proportionality constant between ion flux and pressure gradient (J/ (Pa·s))	5.2×10^{-5}	-
m'	proportionality constant between ion flux and osmolarity gradient (J/ (Pa·s))	5.2×10^{-5}	-
S_{Ep}	Surface area of the renal tubule (m^2)	13.276	[9, 10, 11, 2]
S_{Ed}	Surface area of the endothelial tissue in renal veins (m^2)	0.6	[12]

Table 2: Glossary of model variables and their numerical estimates for the circulatory system. See text for references.

Symbol	Description	Values	Source
R_A	Effective resistance of the aorta (Pa·s/m^3)	7.78×10^7	[5]
R_V	Effective resistance of the inferior vena cava (Pa·s/m^3)	9.98×10^6	[5]
R_O	Effective resistance of the capillaries in all organs (Pa·s/m^3)	1.34×10^8	[5]
R_G	Effective resistance of the glomerulus (Pa·s/m^3)	4.47×10^8	[5]
P_s	Pressure generated by the heart (Pa)	1.6×10^4	[5]
Π_1	Total blood osmolarity (Pa)	8×10^5	[13]
Π_p	Blood oncotic pressure (Pa)	3.8×10^3	[8]

Table 3: Expressions of some effective model parameters. See text for references.

Symbol	Description	Expression
r	dimensionless energy input	$r = \frac{\gamma}{\eta}\frac{\Delta G_a}{RT}$
θ	Model coefficient	$\theta = \frac{r}{r+2}$
Π_0	Weighted mean osmolarity around the pumping element	$\Pi_0 = \frac{(r+1)\Pi_a+\Pi_b}{r+2}$
Π_{02}	Effective osmolarity parameter	$\Pi_{02} = \frac{(r+1)^2\Pi_a-\Pi_b}{r+2}$
α_s	First effective water permeation constant	$\alpha_s = \frac{\alpha}{8\mu L\alpha/R_{cell}^2+2}$
α_{ss}	Second effective water permeation constant	$\alpha_{ss} = \frac{\alpha_s(D+\eta L)}{2\alpha_s\Pi_0+(D+\eta L)}$

References

[1] Jiang H, Sun SX. Cellular pressure and volume regulation and implications for cell mechanics. *Biophys. J.* 2013; 105(3):609-19.

[2] Layton AT, Layton HE. A computational model of epithelial solute and water transport along a human nephron. *PLoS Comput. Biol.* 2019; 15(2):e1006108.

[3] Samson E, Marchand J, Snyder KA. Calculation of ionic diffusion coefficients on the basis of migration test results. *Mater Struct* 2003: 156-65.

[4] Teng X, Huang Q, Dharmawardhana CC, Ichiye T. Diffusion of aqueous solutions of ionic, zwitterionic, and polar solutes. *J. Chem. Phys.* 2018; 148(22).

[5] Kung E, Pennati G, Migliavacca F, Hsia TY, Figliola R, Marsden A, Giardini A, MOCHA Investigators. A simulation protocol for exercise physiology in Fontan patients using a closed loop lumped-parameter model. *J. Biomech. Eng.* 2014; 136(8):081007.

[6] Clausen MV, Hilbers F, Poulsen H. The structure and function of the Na, K-ATPase isoforms in health and disease. *Front. physiol.* 2017 Jun 6;8:371.

[7] Gadsby DC. Spot the difference. *Nature.* 2004; 427(6977):795-7.

[8] Feher JJ. Quantitative human physiology: an introduction. *Academic press.* 2017 Jan 2.

[9] Chevalier RL. The proximal tubule is the primary target of injury and progression of kidney disease: role of the glomerulotubular junction. *Am. J. Physiol. Renal Physiol.* 2016;311(1):F145-61.

[10] Aličelebić S. Proximal convoluted tubules of the rats kidney–a stereological analysis. *Biomol. Biomed.* 2003; 3(1):36-9.

[11] Bertram JF, Douglas-Denton RN, Diouf B, Hughson MD, Hoy WE. Human nephron number: implications for health and disease. *Pediatr. Nephrol.* 2011; 26:1529-33.

[12] Bohle A, Aeikens B, Eenboom A, Fronholt L, Plate WR, Xiao JC, Greschniok A, Wehrmann M. Human glomerular structure under normal conditions and in isolated glomerular disease. *Kidney Int.* 1998; 54:S186-8.

[13] Levenbrown Y, Costarino AT. Edema. Nephrology and Fluid/Electrolyte Physiology. 2019; Elsevier.

Supporting Information Legends

Table S1. Glossary of model variables and their numerical estimates for the pumping element. See text for references.

Table S2. Glossary of model variables and their numerical estimates for the circulatory system. See text for references.

Table S3. Expressions of some effective model parameters. See text for references.
