# Supplementary figures and images for: Fluid and solute transport by cells and a model of systemic circulation
Source: PLoS Comput Biol. 2025 Apr 21;21(4):e1012935. doi: 10.1371/journal.pcbi.1012935 (PMC12040233; doi:10.1371/journal.pcbi.1012935)

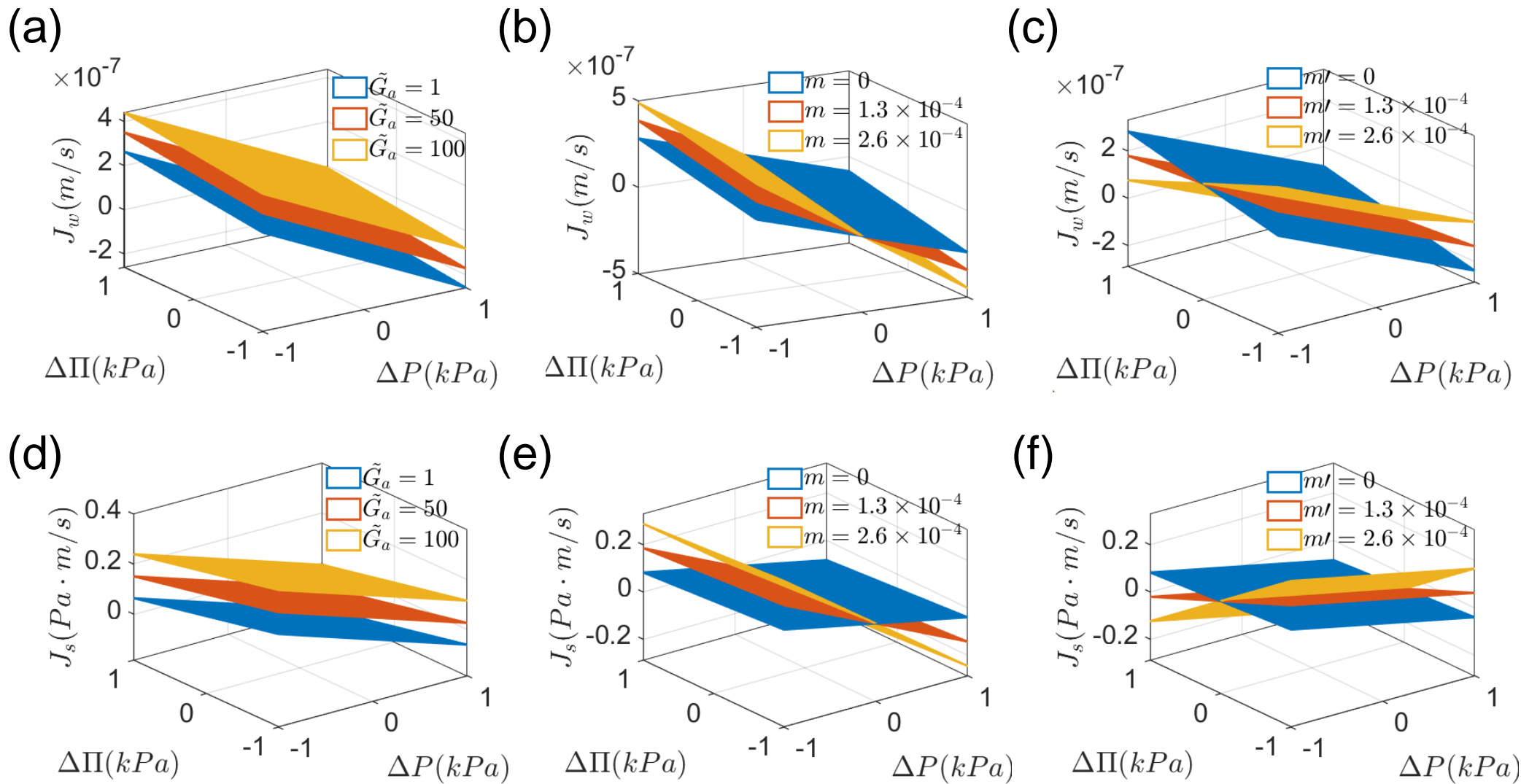

Supplement: S1 Fig — The water and solute fluxes of an isolated pump are determined by basal-apical pressure (ΔP) and osmolarity differences (ΔΠ), energy input (Ga~), and the sensitivity of solute flux to pressure and osmolarity gradients m,m′. The energy input Ga~ increases both the water and solute flux (a&d). In the generalized pump performance surface, m,m′ decrease the slope of the fluxes with respect to pressure gradient ΔP and osmolarity gradient ΔΠ (b,c,e,f). (PDF) [file pcbi.1012935.s003.pdf]

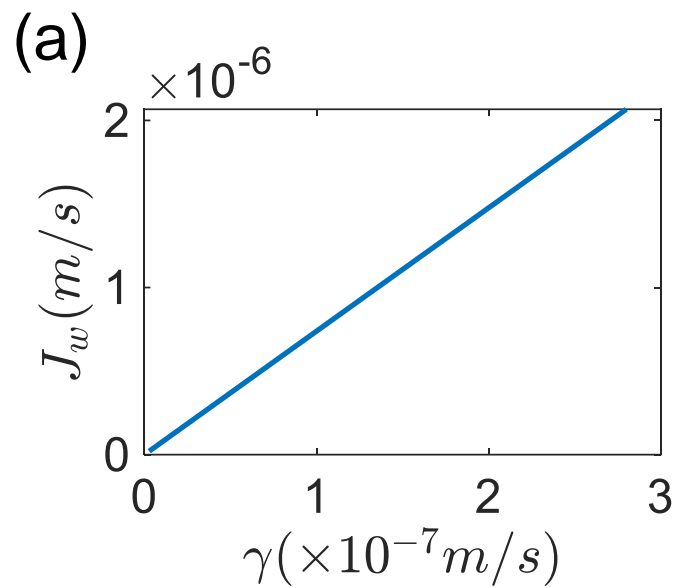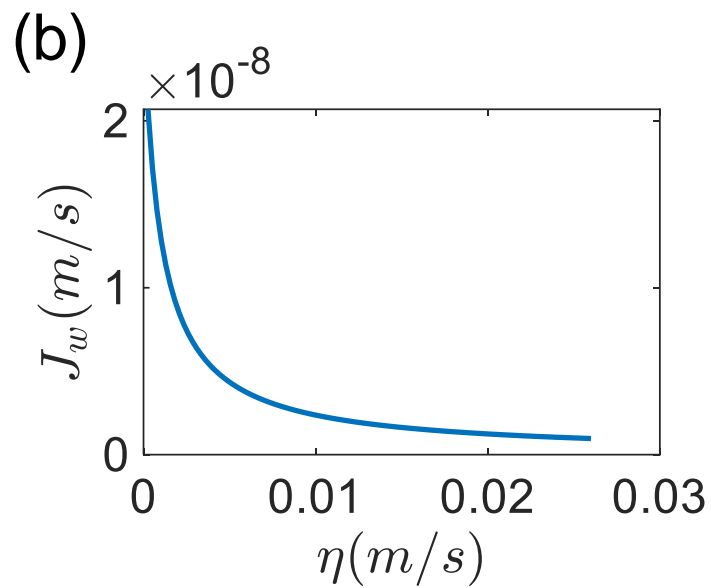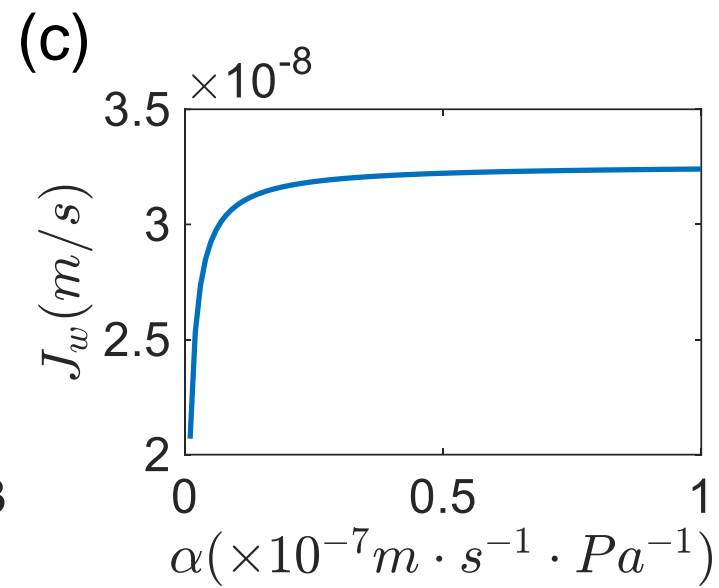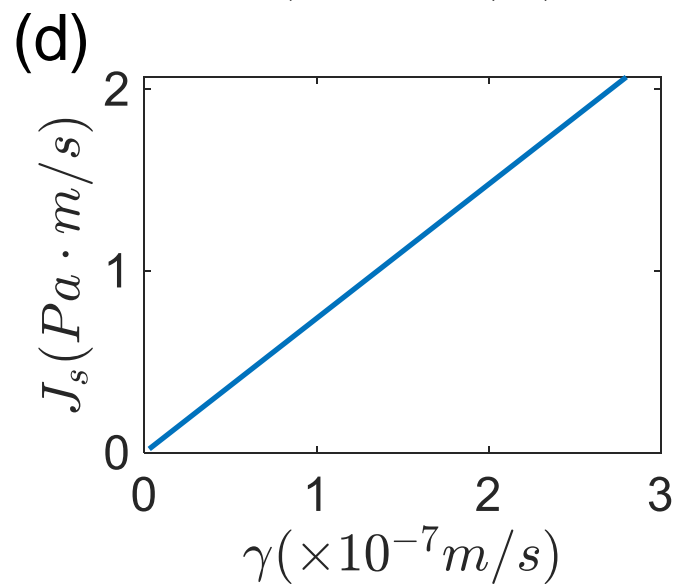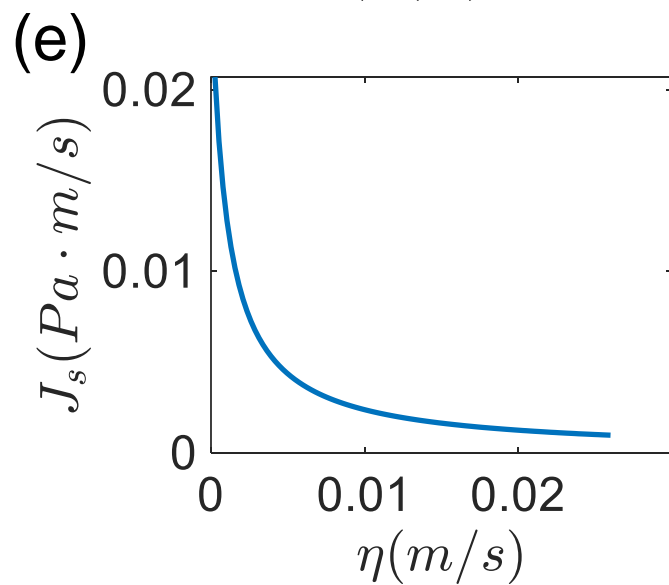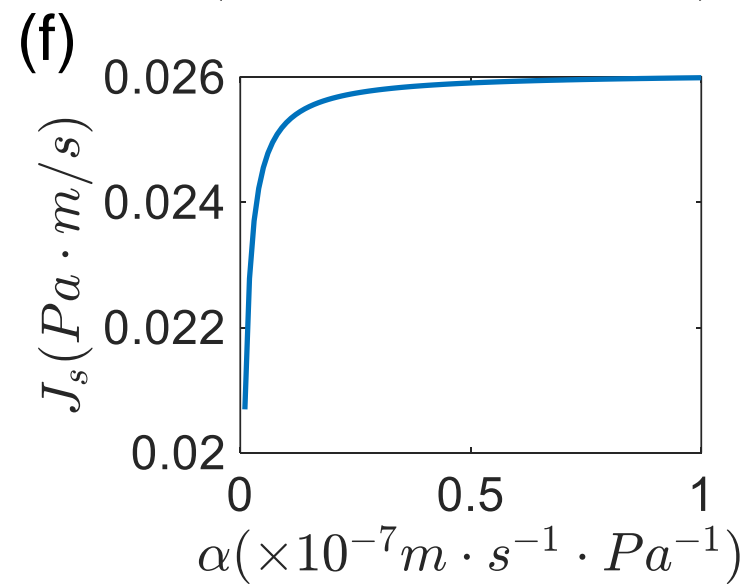

Supplement: S2 Fig — (a) Active transport of ion increases the water flux. (b) Increasing passive transport coefficient decreases water flux. (c) Increase in water permeability leads to increased water flux, reaching a plateau. (d–f) Results on solute flux. (PDF) [file pcbi.1012935.s004.pdf]

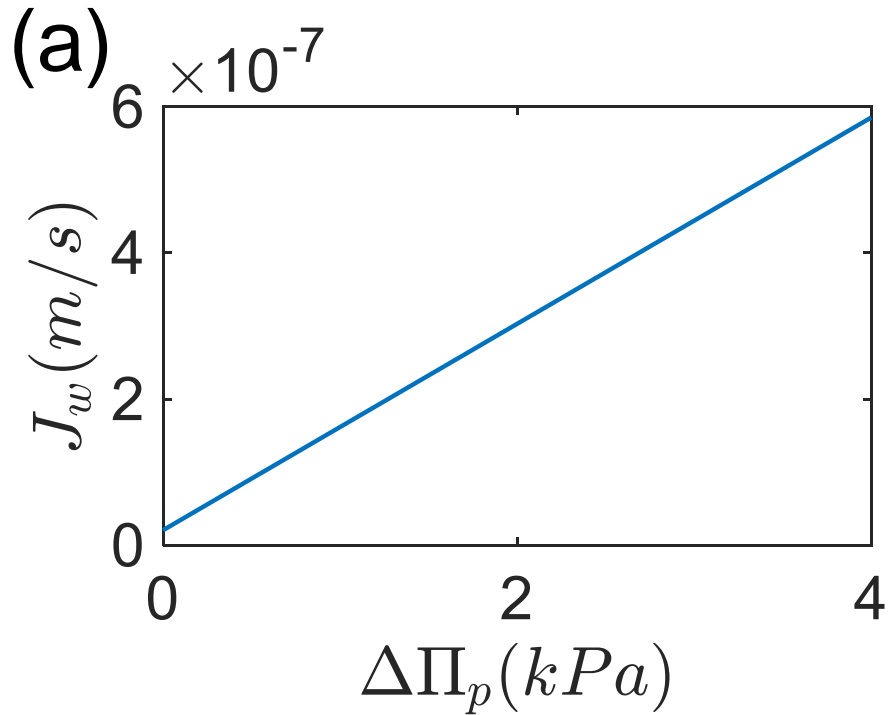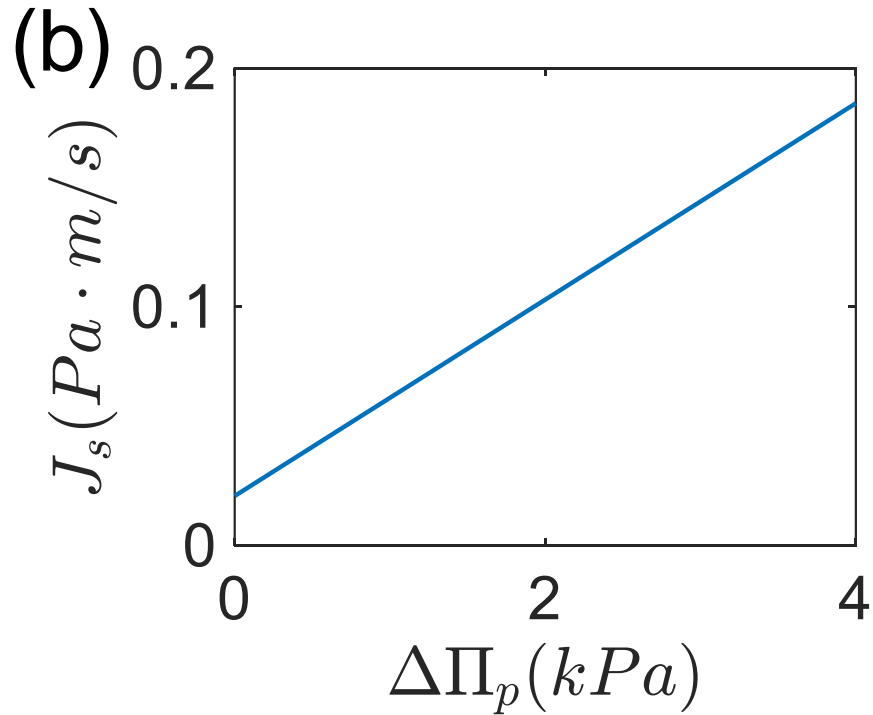

Supplement: S3 Fig — Water and solute fluxes increase with the osmotic pressure gradient ΔΠp, highlighting the role of macromolecule-induced osmotic forces in driving transport across the membrane. (PDF) [file pcbi.1012935.s005.pdf]

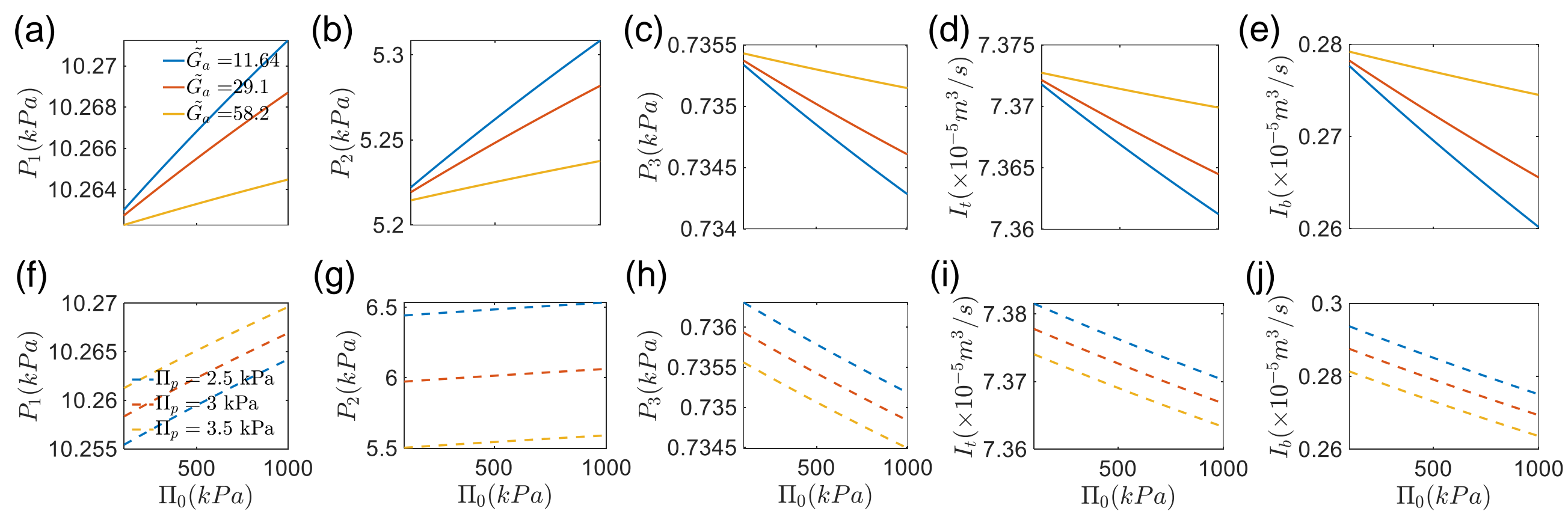

Supplement: S4 Fig — (a)–(c) Increase of the external osmotic pressure causes decrease in pressure at node 3 and increase in node 1 and 2. Increase of energy input decreases the pressure at node 1 and 2 while increases the pressure at node 3. (d)–(e) Total blood flux and the branch flux across the pump both decrease with external osmolarity while increase with energy input. (f)–(h) Oncotic pressure in blood plasma increases the pressure at node 1 while decreases the pressure at node 2 and 3. (i)–(j) Total blood flux and the branch flux across the pump both decrease with the increase of blood oncotic pressure. In (f)–(j), the energy input is set as: G~a=11.64. The water transport constant is set as α=5×10−11 m⋅s−1⋅Pa−1. (PDF) [file pcbi.1012935.s006.pdf]

(a)

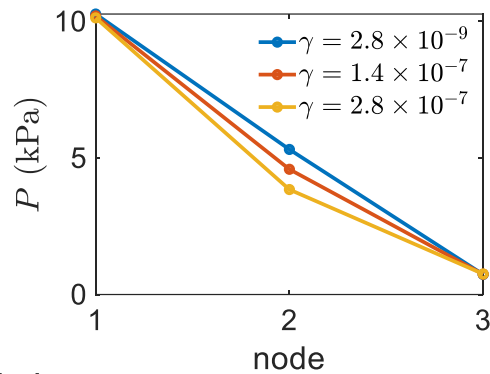

(b)

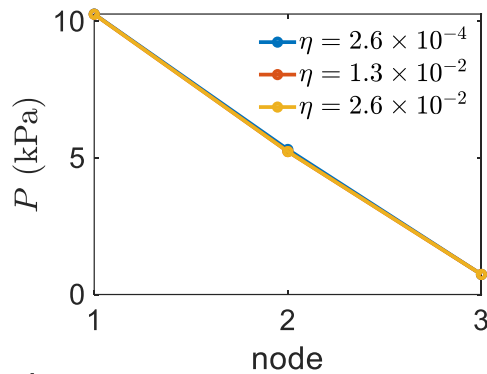

(c)

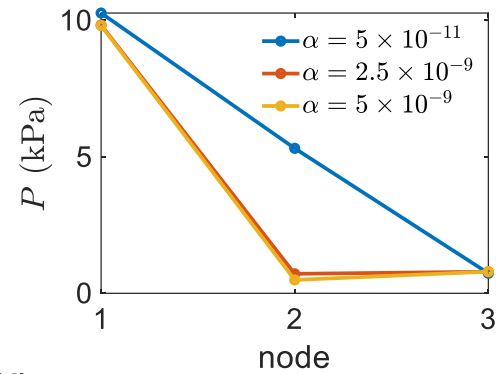

(d)

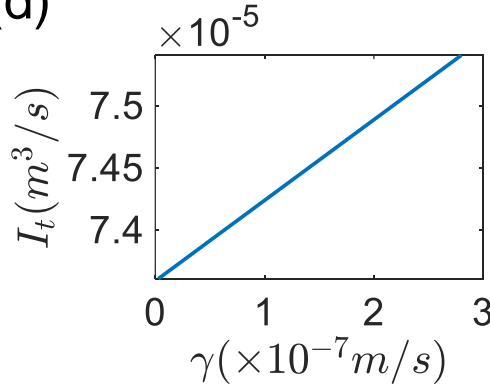

(e)

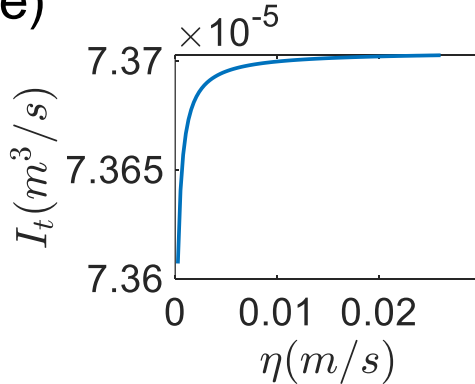

(f)

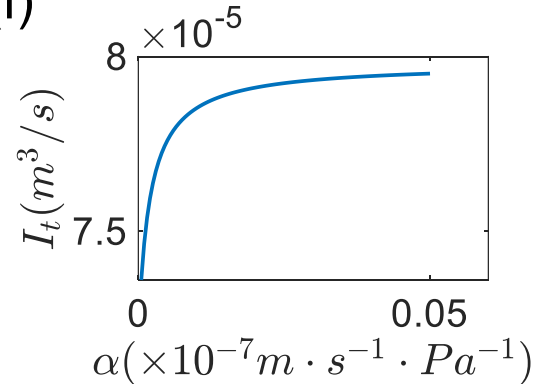

(g)

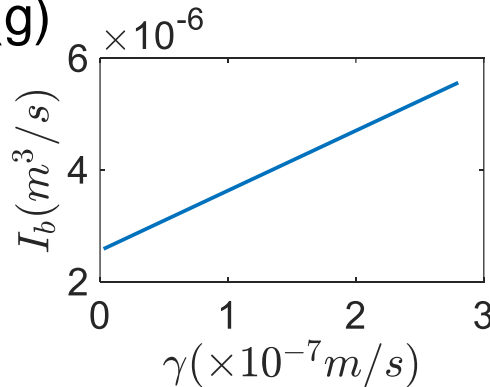

(h)

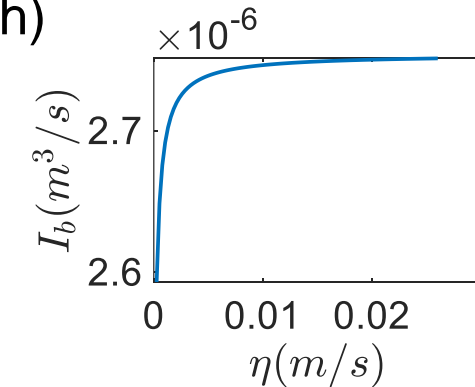

(i)

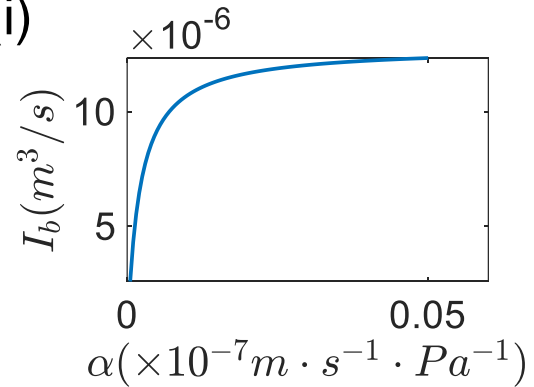

Supplement: S5 Fig — The effects of active, passive ion transport coefficients (γ , η) and water permeability of the membrane (α) on pressure distribution (a–c), total blood flux (d–f) and branch flux (g–i) across the pumping element for the one-pump network are explored. (a)–(c) Both the active ion transport coefficient (γ) and water permeability (α) of the pump decrease the pressure in the lumen of the renal tubule (node 2). (d)–(i) Both the total flux and branch flux across the pump increase with γ , η, and α. (PDF) [file pcbi.1012935.s007.pdf]

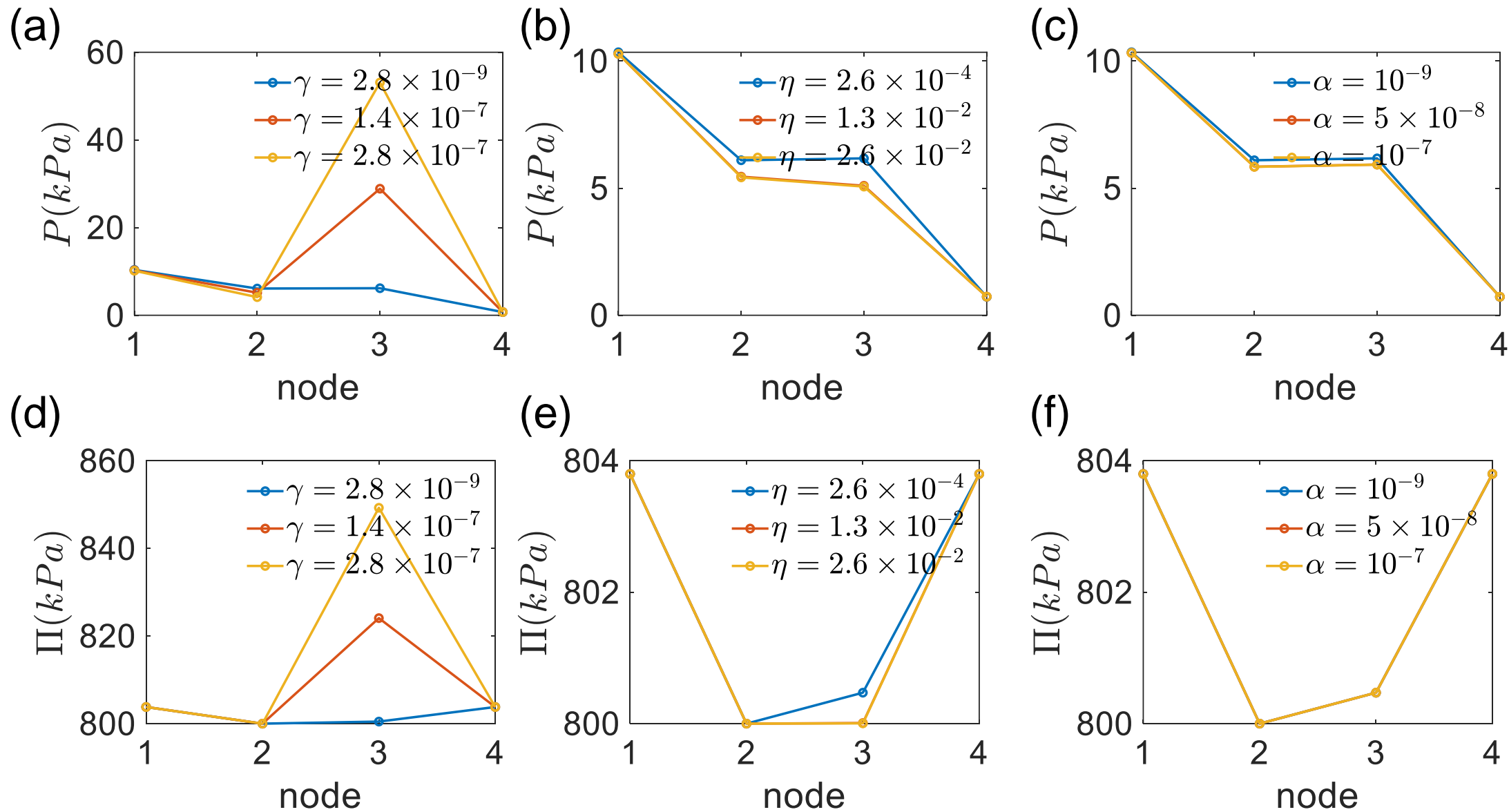

Supplement: S6 Fig — (a)–(c) Influence of active, passive ion transport coefficients (γ , η) and water permeability of the membrane (α) on pressure distribution. The interstitial pressure (node 3) increases with the increase of active ion transport coefficient γ and decrease of passive transport coefficient η and water permeability α. (d)–(f) Results on osmolarity distribution for the two-pump network. The interstitial osmolarity (node 3) increases with the increase of active ion transport coefficient γ and decrease of passive transport coefficient η. (PDF) [file pcbi.1012935.s008.pdf]

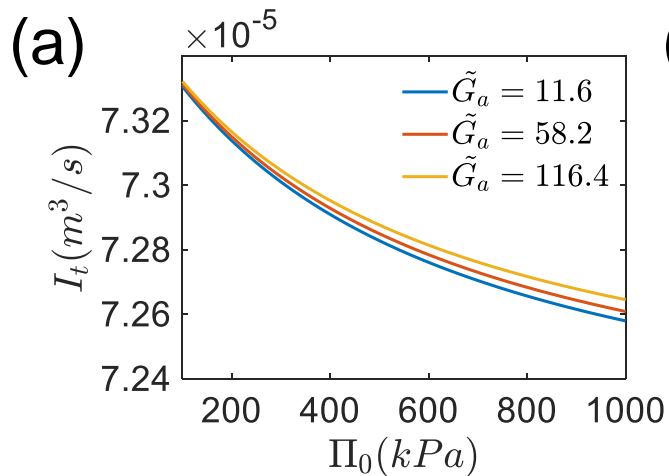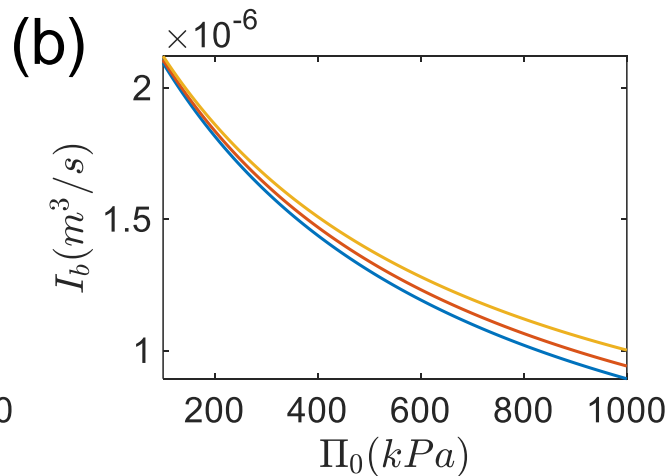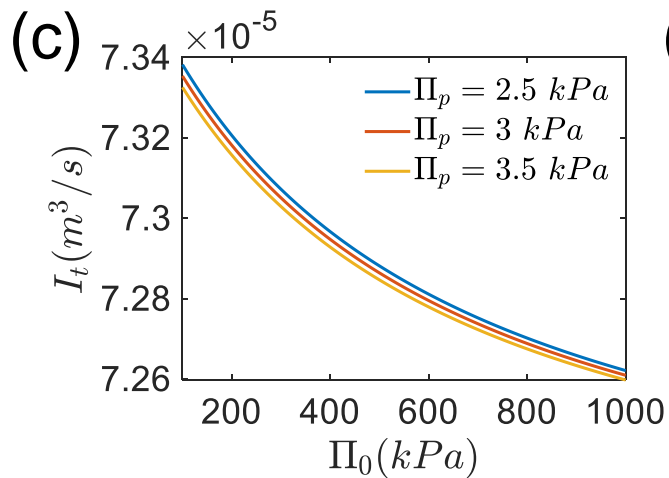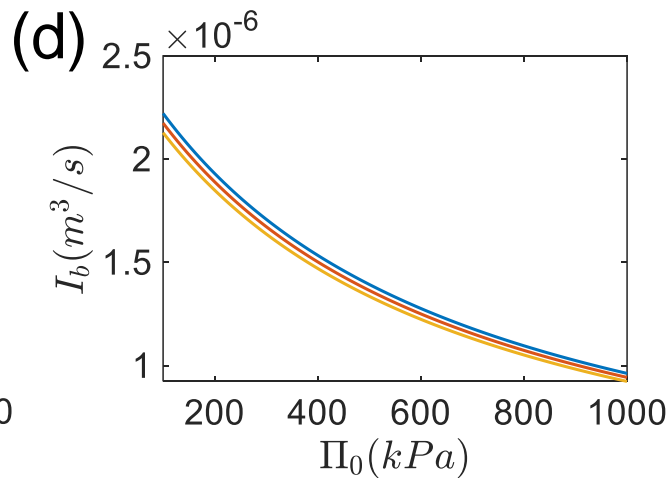

Supplement: S7 Fig — According to the two-pump model, both the total flux and branch flux across the pumping element decrease with elevated external osmotic pressure. (a)–(b) Increase of energy input for the ion pump increases the blood flux. (c)–(d) Increase of blood plasma oncotic pressure decreases the blood flux. (PDF) [file pcbi.1012935.s009.pdf]

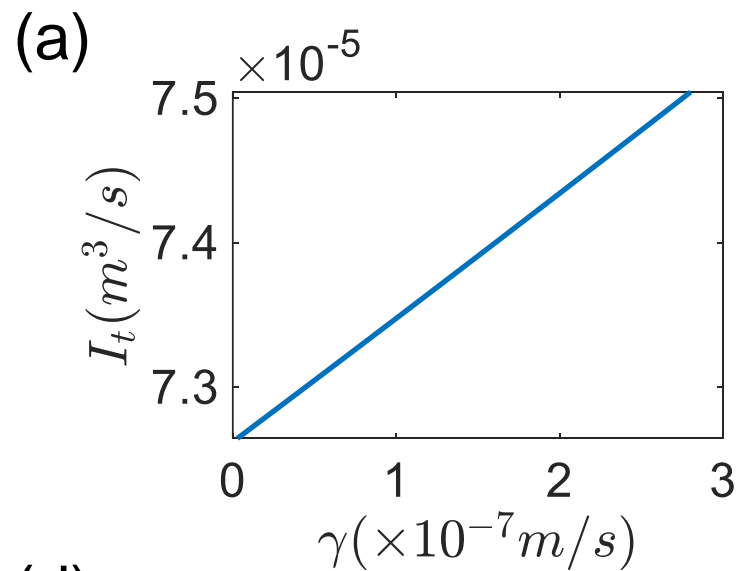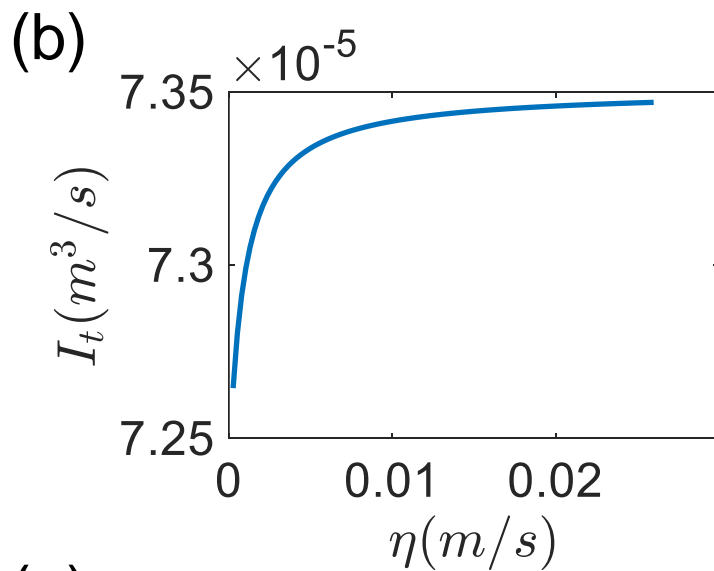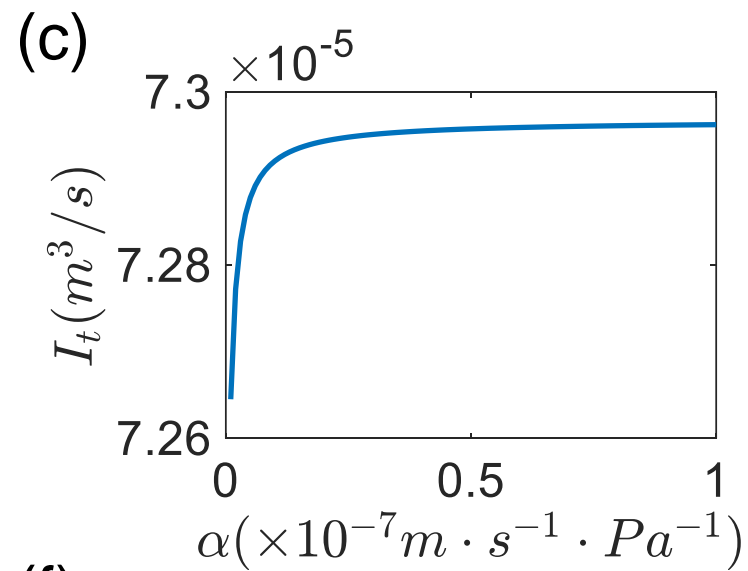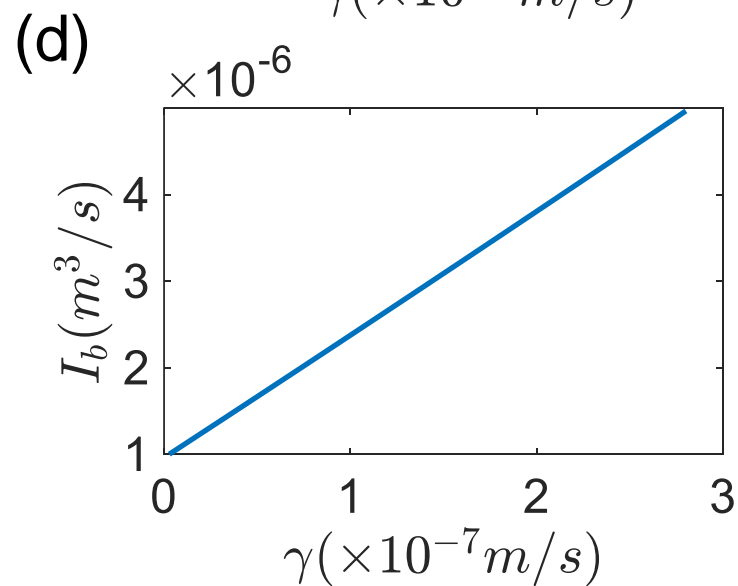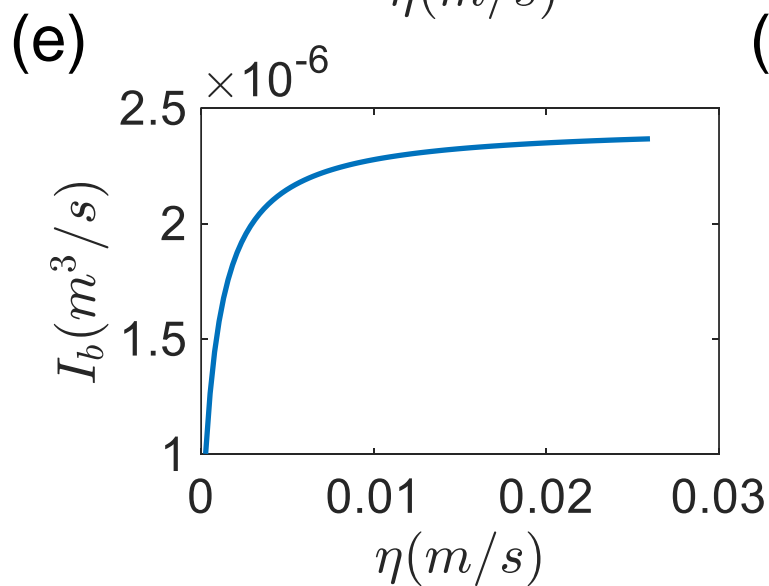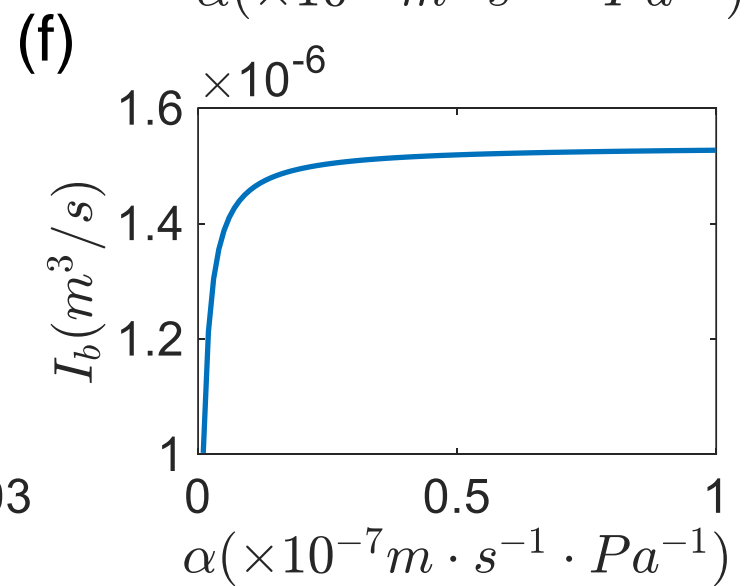

Supplement: S8 Fig — The effects of active, passive ion transport coefficients (γ , η) and water permeability of the membrane (α) on total blood flux (a–c) and branch flux (d–f) across the pumping elements for the two-pump network. (PDF) [file pcbi.1012935.s010.pdf]
