# Supplementary material for: Fluid and solute transport by cells and a model of systemic circulation
Source: PLoS Comput Biol. 2025 Apr 21;21(4):e1012935. doi: 10.1371/journal.pcbi.1012935 (PMC12040233; doi:10.1371/journal.pcbi.1012935)
Supplement: S9 Fig — (a)–(b) spatial distribution of pressure and osmolarity with different energy input, blood oncotic pressure and total osmolarity in blood plasma. (c)–(d) Influence of total blood osmotic pressure, oncotic pressure and energy input on hydraulic pressure and osmotic pressure in the interstitium. When not specified, the energy inputs for kidney epithelial pump and endothelial pump are: (Ga1~,Ga2~)=(29.10,0). All other parameters are the same for both pumps. (PDF) [file pcbi.1012935.s011.pdf]

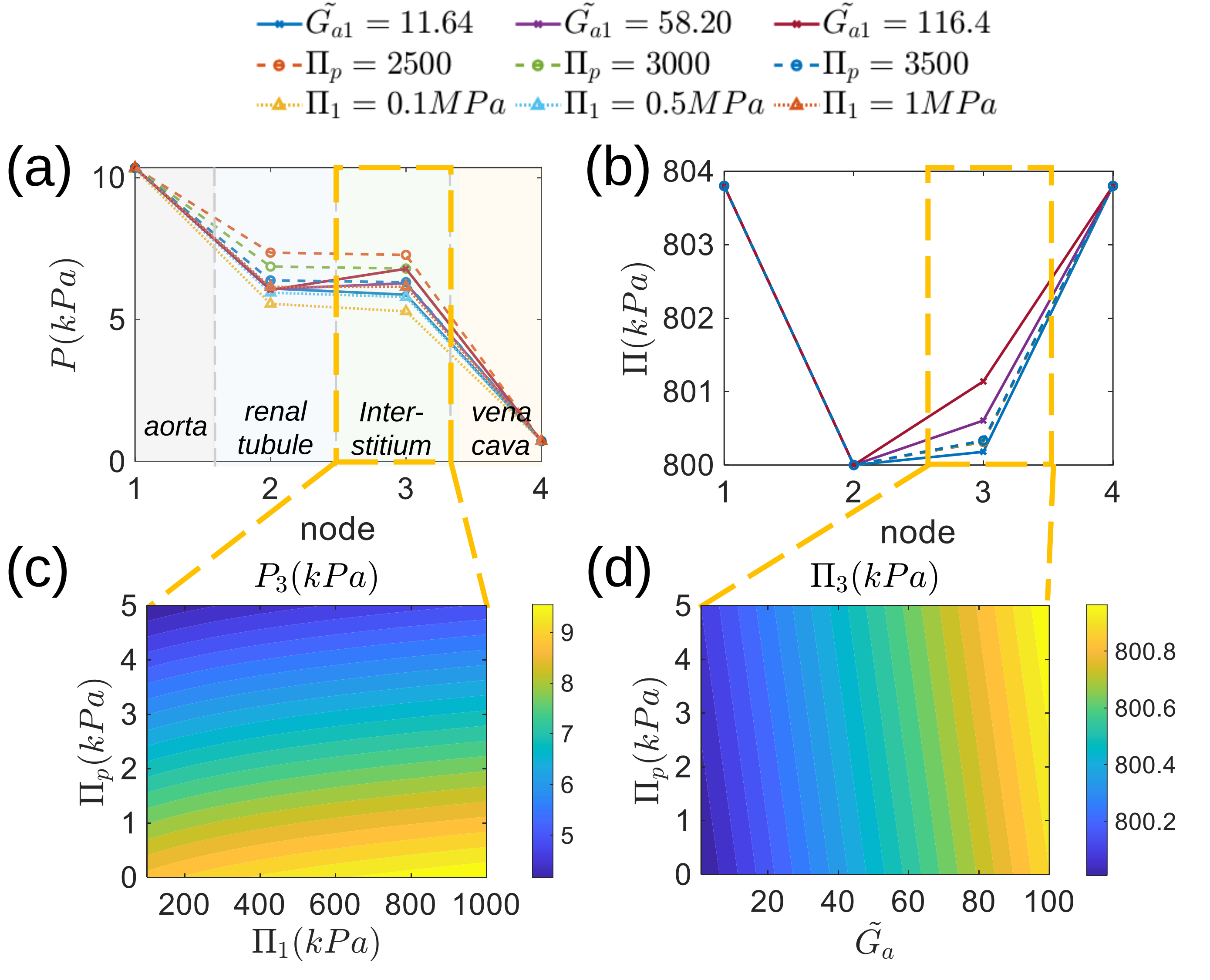

$\tilde{G_{a1}} = 11.64$
$\tilde{G_{a1}} = 58.20$
$\tilde{G_{a1}} = 116.4$
$\Pi_p = 2500$
$\Pi_p = 3000$
$\Pi_p = 3500$
$\Pi_1 = 0.1MPa$
$\Pi_1 = 0.5MPa$
$\Pi_1 = 1MPa$
(a)
$P(kPa)$
10
5
0
aorta
renal tubule
Inter-stitium
vena cava
1
2
3
4
node
(b)
$\Pi(kPa)$
804
803
802
801
800
1
2
3
4
node
(c)
$P_3(kPa)$
$\Pi_p(kPa)$
5
4
3
2
1
0
200
400
600
800
1000
$\Pi_1(kPa)$
9
8
7
6
5
(d)
$\Pi_3(kPa)$
$\Pi_p(kPa)$
5
4
3
2
1
0
20
40
60
80
100
$\tilde{G}_a$
800.8
800.6
800.4
800.2
