# Supplementary material for: Fluid and solute transport by cells and a model of systemic circulation
Source: PLoS Comput Biol. 2025 Apr 21;21(4):e1012935. doi: 10.1371/journal.pcbi.1012935 (PMC12040233; doi:10.1371/journal.pcbi.1012935)
Supplement: Attachment — Submitted filename: Response Letter_PCB_pumping.docx [file pcbi.1012935.s012.docx]

Dear Editor:

We would like to thank you and the reviewers for examining our manuscript. All of the reviewers brought up important concerns and valid points of revision. We have now prepared a revised version according to reviewer suggestions. The changes are highlighted in red in the manuscript.

**Response to Reviewer #1:**

***Comment #1:***

*In this study the authors developed a cellular active filtration mathematical model and coupled it to a simplified lumped parameter model of the circulation. Simulation results show that changes in filtration has effects on bulk blood flow. The filtration model contains a large number of parameters. Since little experimental data currently exists to fully define all of the parameters in the detailed filtration model, the simulation results may be meaningful only qualitatively at the current time, and it is difficult to validate the construction of this model.*

**Response:** We thank the reviewer for the summary and comments. Regarding the first comment on model parameters: In the tissue-level transport model (referred to by the reviewer as the 'filtration model'), the values of all parameters are from literature obtained from experiments. These parameters include ion and water transport rate, energy of ATP hydrolysis and size of cells and tissues. Except for the linear coefficient between ion flux and pressure/osmolarity ($m/m'$), all parameters are obtained from experiments. The linear coefficient between ion flux and pressure/osmolarity ($m/m'$) can be calculated indirectly given the stall pressure (see Choudhury et al., 2022) and active energy input from ATP hydrolysis. Details on these parameter estimation and corresponding sources are explained in the supplemental material in “Supplementary Note 3: Parameter estimation” and “Supplementary Table 1”. We agree that there is some error in measurements and estimation, but we believe that the experiment-based parameters can also provide some reliable quantitative insights besides qualitative prediction in our tissue-level transport model.

Regarding the second comment on model construction, we agree that it is not easy to validate the model predictions in all aspects. However, there is some indirect experimental evidence supporting our major conclusions. For example, one of our major conclusions is that there exists osmolarity gradient inside the cell. This is supported by the fact that epithelial monolayers form domes on substrate (Latorre et al., 2018). This dome formation is actively driven by osmolarity-gradient induced water flux without additional pressure gradient. Also, one of our major assumptions is the dependence of ion flux on pressure and osmolarity, which is based on experimental findings that hydrostatic pressure decreases net sodium transport in rat colonic mucosa (Wanitschke et al., 1977). Therefore, the major part of our model construction is firmly supported by experiments.

***Comment #2:*** *When coupling to the lumped parameter model, there are only a few interface parameters. This presents an opportunity to practically conduct empirical parameter tuning and validation. The qualitative simulation results presented in this study make intuitive sense. There are large degrees of freedom in parameter tuning to obtain desired results. Since there is no validation data for the detailed filtration model, it is difficult to make definitive conclusions regarding the performance of the model presented.*

**Response:** We thank the reviewer for the comment. In the coupled network model, the key parameters are the resistances of various components. All parameter values were adopted from the study by Kung et al. (2013) without additional tuning. Briefly, the resistances were approximated using pipe flow principles described by Poiseuille's law. Given the radius, length, and fluid viscosity, the peripheral resistance for a tube structure can be calculated. The resistances of different components were initially approximated in earlier studies [Noordergraaf et al., 1963; Snyder & Rideout, 1969; Snyder et al., 1968]. Adjustments were made to the resistance of capillary blood vessels, which involve more complex structures. These adjustments were based on real clinical data for systemic pressures and flows, as described in Kung (2013). While these estimates remain approximate, they offer valuable qualitative insights into the behavior of the coupled network model.

**Response to Reviewer #2:**

***Summary:*** *The authors have developed a mathematical model to evaluate fluid flow and hydrostatic and osmotic pressures within a network of ion and fluid pumps. The authors argue that such a network exists in the body insinuating that various epithelial and endothelial elements act as pumping units and their individual performance at the cellular scale gives rise to the steady state flow and pressure variables at the organ scale. Using a simplified circuit, they show that optimal working of the pumps is achieved at the intersection of individual pump performance curves and the system curve of the network.*

**Response:** We thank the reviewer for the accurate and thoughtful summary of our work.

***Comment #1:*** *Minor comments: The plots are highly congested, and legends have very small font size, which makes it hard to read and absorb the paper.*

**Response:** We thank the reviewer for highlighting the issue with the figures. We have rearranged the legends and enlarged the figures to improve clarity and make the plots more readable.

***Comment #2:*** *While the abstract states that the proposed "model generates pressure and osmolarity gradients across physiological compartment”, this in my view is over-representation the model and the findings. The model assumed only two organ systems—heart and the kidney- vascular interface. The conclusions need to be toned down a bit. Furthermore, it must be emphasized that so far there is no evidence that endothelial cells can create hydraulic pressure gradients like epithelial monolayers. Therefore, the addition of endothelial pump in the circuit is rather a conjecture. The references 28 and 29 indicate a ‘pump and leak’ theory in the cornea where the high pressure causes fluid leakage from the lumen rather than active regulation.*

**Response:** We thank the reviewer for the critical insights and constructive feedback. We have toned down the conclusion and emphasized that the conclusion we draw is from our minimal model which features heart and kidney-vascular interface and simplifies other compartments. We have modified the corresponding descriptions in the abstract.

We agree that there are no experimental data directly supporting active fluid transport in endothelium and we have emphasized that this is a conjecture in the main text. Actually, our model can describe both an active pump and a passive filter depending on the active energy input parameter $G_{a}$. The case when the endothelium is viewed as a passive filter is also discussed in the supplementary material in supplementary note 2 and there is no significant difference compared to the case when endothelial cells are active pumping elements (see Supplementary Fig 10). We notice that there is some confusion regarding the definition of a pump. In our model, the “pump” is a general description of active transport of ion and water. Specifically, ions are actively pumped across the cell. Consequently, water flows passively according to the free energy gradient (both pressure gradient and actively established osmolarity gradient). The water flux can be influenced by external hydraulic pressure gradient. However, the pump does not necessarily mean the establishment of hydraulic pressure gradient. It’s when these elements are connected to other components in a network that the pressure gradient is naturally generated. We have added some additional descriptions to clarify our definition of the pump (in Section “Cell monolayer fluid and solute pumping model”). In references 28 and 29, the authors claim that sodium pump (Na/K ATPase) actively transports ions to maintain an osmolarity gradient in order to maintain the thickness and transparency of the cornea. These agree with our basic assumption on the part of active ion transport in the “pumping” model. Given the active ion pumping, the transport of water following pressure and osmolarity gradient is possible, which also rationalizes our conjecture that the endothelial cells can actively pump fluid. We have added additional descriptions emphasizing that this is a conjecture and provided a rationale to support it.

***Comment #3:*** *The result in Fig5d don’t make sense when experimental observations are taken into consideration. Previous work on osmolarity measurements across the renal epithelial tubules shows that water transport happens isosmotically from the tubules into the blood vessels. Here, the calculations give rise to an increasing osmolarity from node 2-4. Can the authors explain this?*

**Response:** We thank the reviewer for bringing up the point on osmolarity gradient. As we mentioned in the main text, the water reabsorption takes two steps: fluid transport from renal tubule to interstitium and transport from interstitium to blood vessel. In the first step of tubule-interstitium transport, most fluid is reabsorbed in the proximal convoluted tubule, where the osmolarity difference between tubule and interstitium is nearly zero. In our model, this can be seen from the small difference in osmolarity between node 2 (tubule) and node 3 (interstitium). This difference is also influenced by the active energy input in epithelial fluid transport ($\tilde{G_{a1}}$). We can see that with a lower $\tilde{G_{a1}}$, we can achieve satisfactory isosmotic condition. However, in the second step from interstitium to blood vessels, this is not isosmotic because the protein concentration in the blood plasma is higher than that of the interstitium. Since we plot the total osmotic pressure, including both ions and macromolecules, we can see a big difference between node 3 (interstitium) and node 4 (Vena cava).

***Comment #4:*** *Assuming the energy input to a pump in biological terms would mean rate of ATP hydrolysis, can the model predict dynamics of optimal energy consumption and the limits around stall pressure? What would that mean in a physiological context?*

**Response:** We thank the reviewer for the insightful question. In the biological context, ATP hydrolysis provides the total energy input ($F$) to the pump, with a specific portion ($\lambda$) allocated to active pumping activity. The energy consumed by the ion pump can be expressed as $G_{a}=\lambda F$, while the remaining energy is dissipated as entropy or heat. In our current model, we assume that a fixed proportion of the energy input is used for ion pumping, implying that $\lambda$ is constant. However, we can definitely include more detailed consumption dynamics, where the proportion of energy consumed ($\lambda$) depends on external environmental factors. Specifically, depending on the ion channel mechanism, $\lambda$ could be a function of the ATP hydrolysis rate ($F$), hydrostatic pressure gradient $\Delta P$ and osmotic pressure gradient $\Delta\Pi$. The mechanical efficiency of converting total energy into mechanical work (water flux) can then be quantified as $\eta\propto J_{w}^{2}/F$, representing the kinetic energy divided by the total energy input. This formulation allows us to identify the state of highest energy efficiency, which could define the regime of optimal energy consumption, as the reviewer suggested. Around stall pressure, water flux is near zero, leading to extremely low mechanical efficiency and this is far from the regime of optimal energy consumption.

We here provide a simple phenomenological model for the energy consumption coefficient $\lambda$. We assume that $\lambda$ decreases with increasing total energy input $F$ and the relation can be expressed as: $\lambda\propto e^{-\theta F}$, where $\theta=0.1$ is a constant. The relative mechanical efficiency as a function of total energy input can be depicted as the following. It is important to note that this assumption regarding the proportion of energy used for active ion pumping ($\lambda$) is purely phenomenological and serves as an illustrative example. The true relationship between $\lambda$ and external environmental factors should ideally be derived from a mechanistic model for the solute transporter or obtained through experimental data. To clarify our discussion on "optimal energy consumption," we have added further elaboration in the last section “Mechanical efficiency and stress in the pumping element”.

**Response to Reviewer #3:**

***Comment #1:***

*The manuscript "Cell-Driven Fluid Dynamics: A Model of Active Systemic Circulation" presents a model of fluid and salt transport across an epithelium, intending to use this tissue-scale model as the basis for simulating fluid circulation in animals with organs like the kidney and intestine. The authors argue that this work is novel in its investigation of an uncharted interaction between osmolarity and pressure within a circulatory system. However, after a thorough review, I find that the manuscript is not yet suitable for publication.*

**Response:** We thank the reviewer for the thorough evaluation of our manuscript.

***Comment #2:*** *Lack of Novelty and Insufficient Literature Review: Fluid transport across epithelial layers is a well-established topic in physiology, with extensive foundational research. The manuscript does not cite crucial literature, making it unclear whether any genuinely novel findings are presented. The coupling between ion transport and pressure has been explored previously in equilibrium contexts. For example, [Marbach and Bocquet (2019)] offer a comprehensive review of osmotic processes and their coupling with pressure, which has already been applied to epithelial contexts (e.g., [Larsen et al., 2000]). If this study’s approach is indeed distinct from existing works, the authors must clarify these similarities and distinctions, specifically addressing how their approach advances current knowledge.*

**Response:** We thank the reviewer for the comment. We have read through the suggested references and added more discussion on these references in the introduction in our revised manuscript. The novelty of our study mainly focuses on the following several points: 1. We emphasize the importance of active characteristics of ion and water transport in renal epithelial tissue. We show how the cells actively establish the osmolarity gradient across themselves. We also characterize the pump performance in analogy to water pumps in engineering; 2. We incorporate tissue-level active pumping model into the circulatory system and emphasis the process of solving both coupled solute circuit (osmotic pressure) and fluid circuit (hydrostatic pressure) together to obtain systemic pressure and flow distribution. The coupling between the two circuits is not well considered in the literature at the systemic level.

***Comment #3:*** *Misattributions and Overlooked Foundational Work: The authors reference Ref. 15 in the manuscript to justify the equation J_w on page 6, which indicates a lack of familiarity with established literature on fluid dynamics across cellular boundaries. The work by [Kay and Blaustein (2019)] offers a historical perspective on the development of this equation in the context of cell volume regulation, and [Fischbarg (2010)] provides a review of fluid transport across epithelial layers. Citing these sources, among others, would help ground the manuscript in the existing body of knowledge.*

**Response:** We thank the reviewer for pointing out these important references. We have read through the references the reviewer mentioned and cited these sources in the main text.

***Comment #4:*** *Ion transport mechanisms within the kidney and intestine have been extensively modeled, with advanced frameworks that incorporate feedback and regulatory processes. For example, the modeling approach described by [Marbach and Bocquet (2016)] on kidney filtration includes a literature review that could serve as a valuable resource. The authors should consult recent studies in this field to better position their model and to highlight any novel contributions, which currently remain ambiguous.*

**Response:** We thank the reviewer for the valuable suggestion. Our network model focuses on the coupling effect of osmotic pressure and hydrostatic pressure on systemic circulation and we do not include the feedback and regulatory process. A primary feedback mechanism regarding ion transport is Tubuloglomerular feedback (TGF), which is a negative feedback regulation of glomerular filtration rate based on the concentration of sodium chloride in the distal tubule. More discussions on the feedback and regulatory processes in kidney filtration have been added in the main text and the discussion.

***Comment #5:***

*The manuscript suffers from poor organization and frequent grammatical errors, which hinder comprehension. Significant revisions are necessary to clarify the logical structure and to enhance readability.*

**Response:** We appreciate the reviewer’s feedback regarding the organization and grammar of the manuscript. We have undertaken substantial revisions to improve the logical structure and enhance readability, ensuring the content is presented clearly and effectively.

***Comment #6:***

*To strengthen the manuscript, the authors should distinguish between established experimental inputs and hypothetical assumptions. For instance, the observation cited in Ref. 3, suggesting pump reorganization with increased pressure, is intriguing. The manuscript could benefit from focusing on this specific experimental insight or, alternatively, providing a broader justification for the validity of this observation. If this assumption is speculative, the authors might analyze a simplified, toy model to determine the conditions under which such assumptions would hold relevance for real-world systems.*

**Response:** We thank the reviewer for the valuable suggestion. The experimental findings in ref.3 provides a mechanistic explanation for our assumption about the sensitivity of ion transport in response to external pressure (characterized by parameter $m$). We have discussed and emphasized the important role of this pressure-dependent pump reorganization and ion transport in the fluid transport rate (Supplementary fig 2) and mechanical efficiency (Fig. 6a). We have emphasized the relation between this experiment finding on pump reorganization and our assumption about ion transport.

***Comment #7:*** *In summary, this manuscript requires substantial revision to be considered for publication. The authors should comprehensively review relevant literature, improve the clarity of their model’s novelty, address both structural and grammatical weaknesses, and present a more coherent justification of their findings relative to existing knowledge.
References:
1. Marbach, S., & Bocquet, L. (2019). Osmosis, from molecular insights to large-scale applications. Chemical Society Reviews, 48(11), 3102-3144]
2. arsen, E. H., Sørensen, J. B., & Sørensen, J. N. (2000). A mathematical model of solute coupled water transport in toad intestine incorporating recirculation of the actively transported solute. The Journal of General Physiology, 116(2), 101-124
3. Kay, A. R., & Blaustein, M. P. (2019). Evolution of our understanding of cell volume regulation by the pump-leak mechanism. Journal of General Physiology, 151(4), 407-416.
4. Fischbarg, Jorge. "Fluid transport across leaky epithelia: central role of the tight junction and supporting role of aquaporins." Physiological reviews 90.4 (2010): 1271-1290.
5. Marbach, S., & Bocquet, L. (2016). Active osmotic exchanger for efficient nanofiltration inspired by the kidney. Physical Review X, 6(3), 031008.*

**Response:** We appreciate the reviewer’s detailed summary and constructive critique. We have carefully addressed the concerns raised, including reviewing relevant literature, clarifying the novelty of our model, improving structural and grammatical aspects, and providing a stronger justification of our findings in the context of existing knowledge.
